# Evaluation of Self-Directed Learning Readiness Among Medical Students in Sharjah, the United Arab Emirates

**DOI:** 10.7759/cureus.70096

**Published:** 2024-09-24

**Authors:** Emad A Nosair, Mohammad Omar, Ghaith AlWawi

**Affiliations:** 1 Basic Medical Sciences, University of Sharjah, College of Medicine, Sharjah, ARE; 2 General Medicine, University of Sharjah, College of Medicine, Sharjah, ARE

**Keywords:** clinical medical student, life-long learning, preclinical medical student, problem-based learning, self-directed learning readiness

## Abstract

Introduction and aim: Self-directed learning (SDL) has become an essential learning principle in medical curricula and a requirement of the professional accreditation standards. It has been advocated that SDL is an effective learning strategy for medical students to develop competence in knowledge acquisition and in promoting life-long learning. Although it has been presumed that problem-based learning (PBL) improves the learner’s SDL readiness (SDLR), its effects are still debatable. This study aimed to investigate the readiness for self-directed learning readiness (SDLR) among undergraduate medical students in problem-based learning (PBL) and non-PBL programs, examining the influence of gender, academic performance, and program year level.

Methods: This cross-sectional study used a validated instrument, the SDLR instrument (SDLRI), to assess self-directed learning readiness. It consists of 20 items classified into the following four domains: learning motivation (six items), planning and implementation (six items), self-monitoring (four items), and interpersonal communication (four items) based upon a five-point Likert scale. Additionally, demographic data like academic year level, gender, and the student’s final grade from the previous semester were collected. Questionnaires were administered to students in their first, third, and fifth (final) academic year across three different medical colleges in the United Arab Emirates (UAE). One of these colleges adopts a hybrid problem-based learning (PBL) approach, while the other two follow non-PBL programs. A total of 386 responses were collected, with 200 students (51.8%) from the hybrid PBL program and 186 students (48.2%) from the non-PBL programs.

Results: The overall mean SDLR score was 59.0±18.2. In both programs, the high-achieving students scored SDLR of 64.7±18.0 (n=112), which was statistically significantly higher than the eight medium- and low-achieving students, 57.8±17.2 (n=224). However, the overall SDLR score showed no significant difference in gender or between PBL and non-PBL programs. Although the SDLR increased as PBL students advanced in the program, it decreased in the non-PBL curricula. However, this difference was not statistically significant.

Conclusion: The high-achieving students displayed a higher level of SDLR than 10 of the medium- or low-achieving students, regardless of the educational program. This highlights the essential effect of the intrinsic factor for undertaking SDL and implies that students should initiate the process. Moreover, enhancing personal development and SDL educational practices might be effective in improving SDLR if partly implemented in medical programs.

## Introduction

Over the past decades, self-directed learning (SDL) has been one of the most active areas of inquiry within adult education and learning. In the original work of Knowles, SDL is defined as a process in which individuals take the initiative, with or without the help of others, in diagnosing their learning needs, formulating learning goals, identifying human and material resources for learning, choosing, and implementing appropriate learning strategies, and evaluating learning outcomes [1]. Brookfield describes the concept of self-teaching and defines it as the assumption of the learner for planning and directing the course of learning [2]. Loeng added that the learner’s aptitude to engage in SDL is a key factor affecting lifelong learning abilities [3].

Self-directed learning is valued as a crucial attribute for health professional graduates due to its numerous benefits. It empowers learners to tackle problem-solving and develop lifelong learning abilities [4]. This approach enables medical graduates to continually enhance their skills, staying relevant in the evolving healthcare landscape across diverse scenarios [5,6]. In addition, self-directed learning enhances learners’ self-management, self-monitoring, and motivation abilities, and develops their ability to collaborate with peers and see them as learning resources [7,8]. Finally, it allows them to engage in learning activities that focus on practicing communication [9]. Moreover, SDL functions as an acquired skill where a learner can demonstrate, with confidence, the ability to apply and refine the skills learned at the workplace [1,10]. Therefore, there is increasing recognition of the importance of SDL within higher education, as evidenced by the recommendations raised by the professional accreditation standards, which aim to develop the skills of self-directed lifelong learning for undergraduate students [11,12]. However, despite the benefits of SDL skills, learners in different contexts lack the opportunity to practice SDL activities [13].

Literature review

Self-Directed Learning Readiness

Because the intrinsic element of undertaking SDL requires individuals to initiate the process, educators have been interested in knowing whether individuals are ready for SDL. Wiley defines SDL readiness (SDLR) as the degree to which the individual possesses the attitudes, abilities, and personality characteristics necessary for SDL [14]. This definition implies two assumptions about SDLR. First, adults are inherently self-directing, i.e., readiness for SDL exists along a continuum and is present in individuals at varying degrees. Second, competencies and readiness required for self-direction can be developed to some extent, and the most effective way to develop SDL is by actively practicing the skills it requires while learning.

Approaches Affecting and Enhancing SDLR

Previous literature described various approaches educators have applied to develop and emphasize students' SDL abilities and readiness. Most importantly, implementing problem-based learning (PBL) in medical programs. Inquiry-based PBL curricula rely on active learning and are closely linked to SDL as a foundational principle, emphasizing it not only as a learning process but also as a crucial outcome [15]. Literature has demonstrated that applying PBL motivates learners to become more active, engaging more actively in their learning process rather than passively absorbing information [16]. It also enhances their ability to pursue lifelong learning [17]. Aziz et al. revealed that applying PBL with or without lecturing enhances the SDL skills better than in the non-PBL teaching method [18]. Frambach et al. documented that the application and acceptance of PBL vary universally due to local cultural factors, such as uncertainty, tradition, and hierarchy, and that the traditions might pose a challenge in SDL compared to Asian and Western students [19]. Other than PBL, team-based learning (TBL), flipped classrooms, online learning, and using targeted SDL educational activities are additional fostering approaches [20-23]. Moreover, internet technologies in higher education, such as WEB folios, showed a motivating influence on the learners’ SDLR [24]. Likewise, the educational platform YouTube has been found to have a vital role in encouraging SDL among learners in the United Arab Emirates (UAE) [25].

Greveson and Spencer have mentioned that SDL readiness depends on the learners' intrinsic characteristics, the nature of the institutional curriculum - whether conventional or innovative - and the motivational influence of the educational environment designed to support SDL [6]. Learning motivation is considered the most influential factor in learners’ SDLR and can be used as a predictor of SDLR [26,27]. Furthermore, other factors include age, gender, academic level, academic achievement, learning approach [28], learning style, method of instruction, and discipline taught [29]. Conversely, previous literature has shown considerable variation in how a learner's gender might affect their SDLR [30,31].

Benefits of Measuring SDLR

In PBL programs, measuring SDLR might help educators utilize and improve the learning experiences that gradually develop SDL skills to suit the assigned PBL curriculum better. Additionally, measurements could be used to identify and supervise students who struggle to cope with PBL environments. Their academic progress could be monitored by repeating the measurement. Regardless of a student’s educational background, the structured and continual evaluation of SDLR will undoubtedly lead medical graduates to be highly employable in the healthcare sector [32]. It is worth noting that learners with insufficient exposure to SDL instructions in the medical program are likely unable to practice the skills required for their life-long learning [33]. Graduating students with heightened SDL aptitude is an essential outcome a medical education provider can offer to the professional employment market. Thus, repeated measuring of SDLR at various stages in medical programs may be essential to ensure that the learning outcomes have been achieved.

Rationale and Importance of the Study

Although the College of Medicine, University of Sharjah (COMUOS) has adopted a hybrid PBL curriculum and SDL strategy since its foundation in 2004, measuring students' readiness for SDL and examining the impact of various influencing factors have never been performed in this college. This study measured SDLR in a medical PBL-based program in the UAE and compared the results with non-PBL programs, while also considering factors that may influence it. The significance of this study lies in encouraging teachers and program designers to mentor students’ readiness for SDL and, as a result, incorporate diverse independent educational activities and methods that foster the adoption of SDL skills. This approach helps develop learners' skills, preparing them to be self-directed and lifelong professionals.

This study aimed to investigate the self-directed learning readiness (SDLR) of undergraduate medical students in the hybrid PBL program (COMUOS), as well as in other non-PBL programs in the UAE, and to examine the relationship between gender, academic achievement - as indicated by the final year grade - and the year level of students and their SDLR score. Research questions are as follows: does the overall SDLR score of students in PBL settings differ from that in non-PBL? Do practicing PBL activities during the program affect the SDLR of students? Moreover, is there a relationship between gender and academic achievement and the SDLR score?

## Materials and methods

Participants

This observational comparative cross-sectional study was conducted in the fall semester of 2019-2020 in the College of Medicine, University of Sharjah (COMUOS). The sampling students were from three different medical colleges as follows: (1) COMUOS, (2) Ras Al-Khaimah Medical and Health Sciences University (RAKMHSU), and (3) Dubai Medical College (DMC). Table 1 summarizes the characteristics of the medical colleges included in the study at the time of data collection.

**Table 1:** Characteristics of the medical colleges included in the study at the time of data collection. COMUOS: College of Medicine, University of Sharjah; RAKMHSU: Ras Al-Khaimah Medical and Health Sciences University; DMC: Dubai Medical College; PBL: problem-based learning

Medical college	COMUOS	RAKMHSU	DMC
Curriculum applied	Hybrid PBL, integrated, and student-centred	System-and lecture-based, and PBL-free	
Program duration	6 years	5 years	5 years
Students‘ gender	Co-education	Co-education	Only females

Inclusion criteria included first-, third-, and fifth-year medical students from the three colleges to allow for a proper insight into the trends of SDLR throughout the years. Other colleges were excluded due to a lack of convenience for the recruiters.

In total, 410 students were handed the instrument, 390 accepted to be enrolled, and four were disposed of due to incompleteness, yielding 386 as the final participants' count with a response rate of 94.1%. The participants were chosen from first-, third-, and fifth-year batches of each college. All the students in the PBL curriculum (200) were from COMUOS, whereas 186 were following non-PBL curricula. The numbers of males and females were 97 (25.9%) and 277 (74.1%), respectively.

Fifty students did not specify their final last year grade, 28 from the PBL program, and 22 from the non-PBL programs. Additionally, 12 students did not specify their genders, 10 from the PBL curriculum and two from the non-PBL curricula.

Instrument

The questionnaire was divided into two parts. The first part consists of demographic data of the students, e.g., gender, year level in the program (early in year one, middle in year three, and final in year five), and the grade of the final last year exams. Students with a final last year grade of ≥90% were defined as high-academic achieving students, while the rest were grouped as medium- and low-achieving students. The second part of the questionnaire was the SDLR instrument (SDLRI) developed and validated by Cheng et al. (appendix) [34]. This instrument consists of 20 items categorized in the following four dimensions of SDL abilities: learning motivation (six items), planning and implementation (six items), self-monitoring (four items), and interpersonal communication (four items). The metric is based upon a five-point Likert scale (from 1 = strongly disagree to 5 = strongly agree), and the SDLRI score ranges between 20 and 100, where higher scores indicate higher levels of SDL abilities. All items of SDLI are positively stated. However, in the current study, neutral responses on the Likert scale, e.g., “3” were not considered in the analysis. Before the study, the instrument was pilot-tested on 25 respondents. Despite the availability of many SDLR scales in literature, the SDLR Instrument was selected in the current study for many reasons as follows: first, it can be used in medical disciplines involving students and healthcare professionals; second, it was developed using the confirmatory factor analysis that verified the framework of SDL rather than using the exploratory analysis; and third, its Cronbach’s alpha coefficient was 0.916 [34]. Finally, Cadorin et al., in their comprehensive systematic review, admitted its validity, reliability, and excellent methodology in estimating the psychometric properties [35].

Data collection

Before data collection, ethical approval was obtained from the Research Ethics Committee of the University of Sharjah, and access to the other two colleges was granted with the approval of their deans. No data of any kind were taken from the universities’ records. The instrument was anonymously distributed without identifiers (e.g., name, university ID, etc.). The participants signed written consent for acceptance to be enrolled in the study.

Data entry and analysis

Data were entered and analyzed using Microsoft Excel 2019 (Redmond, WA: Microsoft Corp.) and SPSS 23.0 (Armonk, NY: IBM Corp.). Study variables were summarized by running descriptive statistics. An independent t-test was used to examine the difference in SDLR mean scores between PBL and non-PBL students, males and females, and high-achieving and medium- and low-achieving students. ANOVA was used to assess the difference in SDLR mean scores between first-, third-, and fifth-year students in both programs and among the domains of the instrument. The mean difference was considered statistically significant for p-value <0.05. Mean scores of the three scales were compared across the three groups of students using a Kruskall-Wallis test. SDLR scores of these students were correlated with their latest final-year scores, including all components of the final examination (2018-2019).

## Results

The overall SDLR mean score was 58.9±18.2 and is considered an average level of readiness toward SDL. The mean scores for the four domains - learning motivation, planning and implementation, self-monitoring, and interpersonal communication - were 20.4±6.7, 14.9±6.9, 11.3±5.5, and 12.4±5.1, respectively. The mean score per item was 2.95 out of 5.00. Table 2 summarizes the demographic data for the participants.

**Table 2:** The demographic data of the entire sample. PBL: problem-based learning

Variable	PBL group		non-PBL group		Total	
	n	%	n	%	n	%
Gender (n=374)						
Male	72	37.9	25	13.6	97	25.9
Female	118	62.1	159	86.4	277	74.1
Total	190	100	184	100	374	100
Academic year (n=386)						
1st year	117	58.5	75	40.3	192	49.7
3rd year	45	22.5	53	28.5	98	25.4
5th year	38	19	58	31.2	96	24.9
Total	200	100	186	100	386	100
Academic achievement (n=336)						
High achievers (≥90%)	62	36	50	30.5	112	33.3
Low achievers (<90%)	110	64	114	69.5	224	66.7
Total	172	100	164	100	336	100

Readiness toward SDL and academic achievement

Table 3 reveals that high-achieving students had a significantly higher score in the total readiness for SDL than their medium- and low-achieving peers across both educational programs studied, with notable differences in the “planning and implementation” and “self-monitoring” domains.

**Table 3:** The SDLR score in relation to the academic achievement of the students. *P value of 0.001 is significantly different from the medium- and low-achieving students. **P value of 0.000 is significantly different from the medium- and low-achieving students. SDLR: self-directed learning readiness

Grade group	Medium- and low-achieving students (<90%)			High-achieving students (≥90%)			Total		
	n (%)	Mean (±SD)	Range (min.-max.)	n (%)	Mean (±SD)	Range (min.-max.)	n (%)	Mean (±SD)	Range (min.-max.)
Total SDLR score	224 (66.7%)	57.8 (17.2)	13-98	112 (33.3%)	64.7 (18.1)*	22-96	336 (100%)	60.1 (17.8)	13-98
Learning motivation	224 (66.7%)	20.4 (6.4)	2-30	112 (33.3%)	21.4 (6.8)	2-30	336 (100%)	20.8 (6.5)	2-30
Planning and implementing	224 (66.7%)	14.3 (6.8)	0-30	112 (33.3%)	17.0 (7.0)*	4-30	336 (100%)	15.2 (6.9)	0-30
Self-monitoring	224 (66.7%)	10.8 (5.4)	0-20	112 (33.3%)	13.2 (5.0)**	0-20	336 (100%)	11.6 (5.4)	0-20
Interpersonal communication	224 (66.7%)	12.4 (5.0)	0-20	112 (33.3%)	13.1 (4.9)	0-20	336 (100%)	12.6 (5.0)	0-20

Readiness toward SDL in PBL and non-PBL/conventional programs

Table 4 shows no significant difference between the two educational programs regarding the students' overall SDL readiness scores and scores across different domains.

**Table 4:** Readiness toward SDL in PBL and non-PBL/conventional programs. SDLR: self-directed learning readiness; PBL: problem-based learning

Educational program	PBL			Non-PBL/conventional			Total		
	n (%)	Mean (±SD)	Range (min.-max.)	n (%)	Mean (±SD)	Range (min.-max.)	n (%)	Mean (±SD)	Range (min.-max.)
Total SDLR score	200 (51.8%)	57.9 (18.4)	13-98	186 (48.2%)	60.0 (17.9)	18-95	386 (100%)	58.9 (18.2)	13-98
Learning motivation	200 (51.8%)	20.1 (6.7)	3-30	186 (48.2%)	20.8 (6.7)	2-30	386 (100%)	20.4 (6.7)	2-30
Planning and implementing	200 (51.8%)	14.8 (6.9)	0-30	186 (48.2%)	15.0 (7.1)	2-30	386 (100%)	14.9 (7.0)	0-30
Self-monitoring	200 (51.8%)	11.1 (5.4)	0-20	186 (48.2%)	11.5 (5.7)	0-20	386 (100%)	11.3 (5.5)	0-20
Interpersonal communication	200 (51.8%)	12.0 (5.0)	0-20	186 (48.2%)	12.7 (5.1)	0-20	386 (100%)	12.4 (5.1)	0-20

Regarding the four domains of the SDLRI

**Figure 1:**
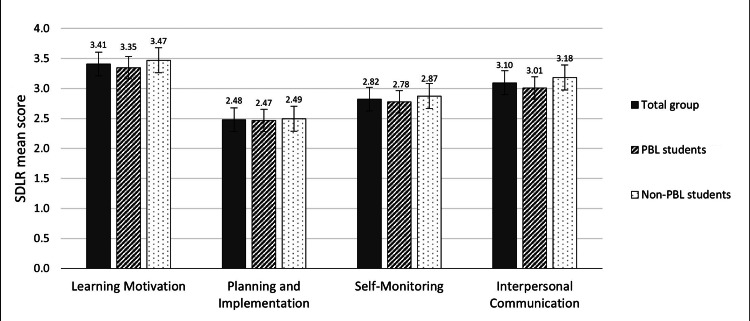
The mean scores of the four domains of the self-directed learning readiness instrument. SDLR: self-directed learning readiness; PBL: problem-based learning

In the current study, the “learning motivation” domain showed the highest mean score (3.41±1.8), followed by the “interpersonal communication” domain (3.10±1.9), then “self-monitoring” (2.82±1.8), and the lowest was the “planning and implementation” domain (2.48±1.8). The same order was observed in the total as well as in the PBL and non-PBL groups. None of the four domains showed a significant difference between PBL and non-PBL groups (Figure 1).

Readiness toward SDL and gender and the progress in the program

Although the 97 male students represented 25.9% of all the sampled students, they showed a slightly higher, non-significant (p=0.639) SDL readiness score (60.1±17.2) compared to females (59.1±18.4). Additionally, in the PBL program, SDL readiness scores increased as students advanced through the years (56.7±17.7 in the first year, 58.5±20 in the third year, and 61.5±18.7 in the fifth year). Conversely, SDL readiness scores decreased in non-PBL programs over the same period (61.6±17.2 in the first year, 59.2±17.3 in the third year, and 58.7±19.4 in the fifth year). However, these changes were not statistically significant (F=0.509, p=0.602). Table 5 shows the SDLR scores of all groups involved in the study and the four domains of the SDL instrument.

**Table 5:** Mean±SD of the SDLR scores of all groups involved in the study and the four domains of the SDL instrument. *P value <0.05 is considered significant. **Independent Student's t-test showed a significant difference between the high achievers and medium/low achievers. ***Multifactorial ANOVA test conducted among the groups in the three academic levels and the domains of the instrument in both programs. SDL: self-directed learning

Variable	PBL program		Non-PBL program		Total		p-Value*
	n (%)	Mean SDLR±SD	n (%)	Mean SDLR±SD	n (%)	Mean SDLR±SD	
Gender (n=374)							
Males	72 (37.9)	59.9 (±16.6)	25 (13.6)	60.4 (±19.0)	97 (25.9)	60.1 (±17.2)	0.639
Females	118 (62.1)	57.7 (±19.3)	159 (86.4)	60.1 (±17.8)	277 (74.1)	59.1 (±18.4)	
Academic achievement (n=336)							
High achievers (≥90%)	62 (36.0)	63.9 (±16.3)	50 (30.5)	65.7 (±20.0)	112 (33.3)	64.7 (±18.0)	0.001**
Medium/low achievers (<90%)	110 (64.0)	56.2 (±18.2)	114 (69.5)	59.4 (±16.2)	224 (66.7)	57.8 (±17.2)	
Year level (n=386)							
Year 1	117 (58.5)	56.7 (±17.7)	75 (40.3)	61.6 (±17.2)	192 (49.7)	58.7 (±18.0)	0.691***
Year 3	45 (22.5)	58.5 (±20.0)	53 (28.5)	59.2 (±17.3)	98 (25.4)	59.3 (±19.0)	
Year 5	38 (19.0)	61.5 (±18.7)	58 (31.2)	58.7 (±19.4)	96 (24.9)	60.1 (±19.0)	
SDLR domains							
Learning motivation	-	20.1 (±6.7)		20.8 (±6.7)		20.4 (±6.7)	0.691***
Planning and implementation	-	14.8 (±6.9)		15.0 (±7.1)		14.9 (±7.0)	
Self-monitoring	-	11.1 (±5.4)		11.5 (±5.7)		11.3 (±5.5)	
Interpersonal communication	-	12.0 (±5.0)		12.7 (±5.1)		12.4 (±5.1)	

Regarding the results of the mean item scores

Table 6 shows the detailed scores of SDLRI’s items (max=5). The overall mean score (±SD) was 2.95 (±1.9), reflecting average readiness towards SDLR which can be improved, PBL group's mean score (±SD) was 2.90 (±1.9), and non-PBL group's mean score (±SD) was 3.00 (±1.9). The overall range was 2.09, in PBL group 1.86, and non-PBL was 2.33, without significant difference between the two medical programs. Out of the 20 item scores, 75% of the items (n=15) showed an average level of readiness that can be improved (2.5-3.5). Three items, all in the “learning motivation” domain, namely 3, 5, and 6 showed a high level of readiness (>3.5), while only three items, all in the “planning and implementation” domain, namely 8, 10, and 11 scored <2.5 which reflects problem areas for all students.

**Table 6:** Mean (±SD) of the self-directed learning instrument item scores (max=5) between PBL and non-PBL programs. PBL: problem-based learning

Item	Total group (n=386)	PBL students (n=200)	Non-PBL students (n=186)
	Mean (±SD)	Mean (±SD)	Mean (±SD)
Learning motivation			
1. I know what I need to learn.	2.56 (±1.9)	2.30 (±1.9)	2.83 (±1.9)
2. Regardless of the results or effectiveness of my learning, I still like learning.	3.08 (±1.9)	3.07 (±1.9)	3.08 (±1.9)
3. I strongly hope to constantly improve and excel in my learning.	4.19 (±1.5)	4.11 (±1.5)	4.27 (±1.4)
4. My successes and failures inspire me to continue learning.	3.48 (±1.8)	3.50 (±1.8)	3.47 (±1.8)
5. I enjoy finding answers to questions.	3.56 (±1.8)	3.62 (±1.7)	3.49 (±1.9)
6. I will not give up learning because I face some difficulties.	3.57 (±1.8)	3.48 (±1.8)	3.67 (±1.7)
Planning and implementation			
7. I can pro-actively establish my learning goals.	2.55 (±1.9)	2.42 (±1.9)	2.70 (±2.0)
8. I know what learning strategies are appropriate for me in reaching my learning goals.	2.46 (±2.0)	2.49 (±2.0)	2.42 (±2.0)
9. I set the priorities of my learning.	2.81 (±2.0)	2.82 (±2.0)	2.81 (±2.0)
10. Whether in the clinical practicum, classroom, or on my own, I am able to follow my own plan of learning.	2.41 (±2.0)	2.43 (±2.0)	2.39 (±2.0)
11. I am good at arranging and controlling my learning time.	2.10 (±1.8)	2.25 (±1.8)	1.94 (±1.8)
12. I know how to find resources for my learning.	2.53 (±2.0)	2.38 (±2.0)	2.70 (±2.0)
Self-monitoring			
13. I can connect new knowledge with my own personal experiences.	3.13 (±1.9)	2.91 (±2.0)	3.37 (±1.8)
14. I understand the strengths and weakness of my learning.	3.02 (±1.9)	3.03 (±1.8)	2.99 (±2.0)
15. I can monitor my learning progress.	2.51 (±2.0)	2.50 (±2.0)	2.53 (±2.0)
16. I can evaluate on my own my learning outcomes.	2.63 (±1.9)	2.66 (±1.9)	2.60 (±2.0)
Interpersonal communication			
17. My interaction with others helps me plan for further learning.	3.18 (±1.9)	3.09 (±1.9)	3.27 (±1.9)
18. I would like to learn the language and culture of those whom I frequently interact with.	3.15 (±2.0)	2.87 (±2.0)	3.45 (±1.9)
19. I am able to express messages effectively in oral presentations.	2.78 (±2.0)	2.86 (±1.9)	2.68 (±2.0)
20. I am able to communicate messages effectively in writing.	3.27 (±1.9)	3.22 (±1.9)	3.33 (±1.9)
Total	59	58	60
Mean (±SD)	2.95 (±1.9)	2.90 (±1.9)	3.00 (±1.9)

## Discussion

The current work revealed an overall moderate level of SDLR in both programs (58.9±18.2). The dominant number of the medium- and low-achieving students (n=224, 66.7%) who exhibited a significantly lower SDLR score could partially explain this moderate level. On the other hand, the high-achieving students who exhibited a significantly higher SDLR score formed the minority of the participants, e.g., only n=112, 33.3%.

Regarding gender, the present study revealed a non-significant higher SDLR score in males - despite their relatively lower number (n=97, 25.9%) - than in females (n=277, 74.1%) regardless of the educational program. This result is consistent with the findings of Phillips et al. [31] and El-Gilany and Abusaad [36]. Nevertheless, the literature showed significant disagreement regarding SDLR and gender. Although most studies reported significantly higher SDLR in females [37,38], others reported significantly higher SDLR among males [30]. A potential explanation for this finding in the current study might be the dominant number of participating females.

Relationship between SDLR and the educational programs

The current study showed no significant difference in SDLR between PBL and non-PBL students. Similar findings were observed by Shaikh between students in the traditional curriculum and those following the integrated organ systems-based curriculum [39]. On the contrary, other studies have found a significant increase in SDLR throughout PBL curricula compared with non-PBL curricula [17,21,40,41]. As a result, the variability in findings across the literature may reflect uncertainty about the relationship between these two variables, or it could be due to different study designs and methodologies.

Leary reported that PBL promotes SDL skills that would be visible easily to an expert and noticeable to a casual observer [42]. Compared to previous meta-analyses focused on cognitive outcomes, Belland et al. have reported low but positive effect sizes favoring PBL [43]. Claims in the literature suggest that PBL promotes SDL skills. Having evidence to support this, alongside positive cognitive outcomes, is beneficial for future PBL research and practice. Moreover, Gerald and Grow advocated that SDL skills could be purposefully integrated into the curriculum through a staged planning model [44]. Although SDL is strongly recommended for integration-based systems, Candy advised implementation in a systems-based approach [15]. It is worth noting that learners with a deficiency in practicing SDL cannot apply its skills in their life-long learning [33].

Relationship between SDLR and progression in the program

The current study found no statistically significant difference in SDL readiness between students from PBL programs and those from non-PBL programs as they advanced from their first to fifth year. Similarly, Williams found that students did not demonstrate any increase in SDLRS at the end of one year in a PBL program [5]. Slater et al. concluded that the passing of time, accumulation of life experience, increasing age, and program progression need time to develop many SDL characteristics and to engage them in a range of life, study, and work experiences [38]. It may be that these factors are alternative measures for more involving concepts such as individual cognitive or social development.

The absence of improvement in the SDLR with progression in the programs might be explained by the fact that some students struggle to engage in SDL because they lack independence, confidence, or resources and that not all adults prefer the self-directed option and prefer formal teacher-directed courses [45]. Likewise, students undergo a transformation that begins with negative feelings but ends with confidence and skills in self-direction, and during this transformation, it is the responsibility of the teachers to provide student support [46]. Frambach et al. stated that the acceptability of SDL skills shows variation across different cultures and countries [19]. Culture and tradition were principal restraints in Middle Eastern students’ SDLR, whereas dependence on hierarchical sources rather than oneself is challenging to SDLR in Asian students. However, these factors had minimal influence on students in Western countries. It was noted, however, that once introduced, students grew accustomed to newer education methods, and acceptability and skills in SDL increased across different cultures despite the various challenges in each setting. Similarly, the impact of culture extends to communication and the learning strategies adopted.

The SDLR domains and individual items

The detailed scores of SDLRI’s items showed that the overall mean score was 2.95 (±1.9), which is considered as an average readiness towards SDLR which can be improved. The lowest score was obtained in only three items, e.g., no. 8 (I know what learning strategies are appropriate for me in reaching my learning goals), 10 (Whether in the clinical practicum, classroom, or on my own, I am able to follow my own plan of learning), and 11 (I am good at arranging and controlling my learning time). These three items belong to the “planning and implementation” domain. These findings highlight the weak areas in the students' SDLR, which require the educators to take corrective actions to enhance the students' time management and planning skills.

Implications and recommendations

Measuring SDLR has many implications to be recommended, such as (1) training teachers to coach students for the SDL skills and implement a student-centered environment with well-structured and student-centered courses and activities, (2) individualizing teaching by classifying students before distributing group assignments according to their level of SDLR. So, students who are not ready with SDL could be coached or placed under the guidance of a more skilled peer, (3) predicting students’ performance in admission to medical colleges, and (4) identifying the weak areas/domains in the students’ SDLR for taking corrective actions, as planning for specific courses or workshops for improvement [44].

Limitations

The limitations include a small sample size with diverse demographic characteristics, as the number of female students, medium/low achievers, and first-year students was disproportionately high. Additionally, using only the final year grade as a measure of student achievement is a limitation.

## Conclusions

The current study concluded that the high-achieving students displayed a significantly higher level of SDLR mean score than the medium/low achievers regardless of the educational approach they follow. However, no significant differences were observed regarding gender, year level, or the educational program. This supports the essential influence of the intrinsic factor for undertaking SDL. Students need to be supported in their SDL skills by fostering a student-centered mindset in all activities of the educational environment.

## Appendices

**Table 7:** The study questionnaire. Responses are rated on a scale from 1 to 5, where 1 = strongly disagree, 2 = disagree, 3 = neutral, 4 = agree, and 5 = strongly agree.

Item	Score				
1. I know what I need to learn.	1	2	3	4	5
2. Regardless of the results or effectiveness of my learning, I still like learning.	1	2	3	4	5
3. I strongly hope to constantly improve and excel in my learning.	1	2	3	4	5
4. My successes and failures inspire me to continue learning.	1	2	3	4	5
5. I enjoy finding answers to questions.	1	2	3	4	5
6. I will not give up learning because I face some difficulties.	1	2	3	4	5
7. I can proactively establish my learning goals.	1	2	3	4	5
8. I know what learning strategies are appropriate for me in reaching my learning goals.	1	2	3	4	5
9. I set the priorities of my learning.	1	2	3	4	5
10. Whether in the clinical practicum, classroom, or on my own, I am able to follow my own plan of learning.	1	2	3	4	5
11. I am good at arranging and controlling my learning time.	1	2	3	4	5
12. I know how to find resources for my learning.	1	2	3	4	5
13. I can connect new knowledge with my own personal experiences.	1	2	3	4	5
14. I understand the strengths and weaknesses of my learning.	1	2	3	4	5
15. I can monitor my learning progress.	1	2	3	4	5
16. I can evaluate on my own my learning outcomes.	1	2	3	4	5
17. My interaction with others helps me plan for further learning.	1	2	3	4	5
18. I would like to learn the language and culture of those whom I frequently interact with.	1	2	3	4	5
19. I am able to express messages effectively in oral presentations.	1	2	3	4	5
20. I am able to communicate messages effectively in writing.	1	2	3	4	5
